# The effect of prior knowledge of color on reaction time depends on visual modality

**DOI:** 10.1016/j.heliyon.2022.e09469

**Published:** 2022-05-20

**Authors:** Takayuki Horinouchi, Tatsunori Watanabe, Takuya Matsumoto, Keisuke Yunoki, Takayuki Kuwabara, Kanami Ito, Haruki Ishida, Hikari Kirimoto

**Affiliations:** a Department of Sensorimotor Neuroscience, Graduate School of Biomedical and Health Sciences, Hiroshima University, Hiroshima, Japan; b Faculty of Health Sciences, Aomori University of Health and Welfare, Aomori, Japan; c Research Fellow of Japan Society for the Promotion of Science, Japan

**Keywords:** Go/No-go task, Reaction time, Prior knowledge of color, Visual modality

## Abstract

Prior knowledge of color, such as traffic rules (blue/green and red mean “go” and “stop” respectively), can influence reaction times (RTs). Specifically, in a Go/No-go task, where signals were presented by a light-emitting diode (LED) lighting device, RT has been reported to be longer when responding to a red signal and withholding the response to a blue signal (Red Go/Blue No-go task) than when responding to a blue signal and withholding the response to a red signal (Blue Go/Red No-go task). In recent years, a driving simulator has been shown to be effective in evaluation and training of driving skills of dementia and stroke patients. However, it is unknown whether the change in RT observed with the LED lighting device can be replicated with a monitor presenting signals that are different from the real traffic lights in terms of depth and texture. The purpose of this study was to elucidate whether a difference in visual modality (LED and monitor) influences the effect of prior knowledge of color on RTs.

Fifteen participants performed a simple reaction task (Blue and Red signals), a Blue Go/Red No-go task, and a Red Go/Blue No-go task. Signals were presented from an LED lighting device (Light condition) and a liquid crystal display (LCD) monitor (Monitor condition).

The results showed that there was no significant difference in simple RT by signal color in both conditions. In the Go/No-go task, there was a significant interaction between the type of signal presentation device and the color of signal. Although the RT was significantly longer in the Red Go/Blue No-go than Blue Go/Red No-go task in the Light condition, there was no significant difference in RT between the Blue Go/Red No-go and Red Go/Blue No-go tasks in the Monitor condition.

It is interpreted that blue and red signals presented from the LCD monitor were insufficient to evoke a perception of traffic lights as compared to the LED. This study suggests that a difference in the presentation modality (LED and monitor) of visual information can influence the level of object perception and consequently the effect of prior knowledge on behavioral responses.

## Highlights

• Prior knowledge of color influences reaction time in a Go/No-go task.
• An LED and an LCD monitor were used as signal presentation devices.
• The signal presentation by the LED influenced reaction time, but not by the LCD.
• The effect of prior knowledge of color on reaction time depends on visual modality.

## 1 Introduction

In our daily lives, we often choose actions based on visual information. Measuring reaction time (RT) is one method of evaluating the level of processing of external stimuli including visual information. RT is defined as the time between the presentation of an external signal and the occurrence of a response to that signal. It can be subdivided into four parts: 1) the initial visual processing time from the retina to the primary visual cortex, 2) visuo-motor related time (VMRT), during which motor commands are generated in the primary motor cortex after processing of information in higher-order visual areas, prefrontal cortex, and parietal association areas [1,2], 3) corticospinal conduction time from the primary motor cortex to the muscle, and 4) electromechanical delay, the time delay between the muscle activity onset and the onset of joint movement. Of these, the VMRT is known to vary depending on the task difficulty [3], such as the complexity of visual information and the degree of cognitive load. Since the other components are expected to be consistent, a change in RT mainly reflects a change in VMRT and thus the visual information processing. For example, in a Go/No-go task, during which participants are required to respond when a target (Go) signal is presented but must refrain from responding when a non-target (No-go) signal is presented, the RT was found to be shorter in professional baseball players than general university students, even though their RTs in a simple reaction task were similar [4]. It was interpreted that this difference was caused by superior information processing and decision-making ability to external stimuli by long-term practice in professional baseball players. Moreover, brain activity associated with response conflict and movement inhibition was revealed to be larger in the No-go than Go trial [5, 6]. These behavioral and neurophysiological data indicate that RT in the Go/No-go task is related to cognitive information processing associated with execution and inhibition of a motor response [7].

The execution and inhibition of voluntary movements are often influenced by the meaning of color in context-relevant situations. For example, in our daily lives, we determine our actions based on the meaning of colors, such as traffic lights and warning signs. An international standard for the meaning of colors for safety signs has been established by the International Organization for Standardization (ISO): “Graphical symbols – Safety colours and safety signs – Registered safety signs (ISO 7010)” [8]. In the ISO 7010, red means “Prohibition” and blue means “Must do,” and our actions are often selected according to these meanings. There are some studies that have investigated the effect of color and its meaning on RTs. Although simple RTs to blue and red lights were found be similar [9], several studies show that RTs can be affected when the meaning of color is manipulated. For example, RTs have been shown to be prolonged when responding to a pedestrian traffic light, signaling “stop” presented in a blue color [10]. Furthermore, in a Go/No-go task using a light-emitting diode (LED) lighting device, RTs were found to be longer when responding to a red “Go” signal and withholding the response to a blue “No-go” signal (Red Go/Blue No-go task) than when responding to a blue “Go” signal and withholding the response to a red “No-go” signal (Blue Go/Red No-go task) [11]. As brain activity reflecting response conflict [12, 13, 14] was larger in the Red Go/Blue No-go than Blue Go/Red No-go task, the finding of RT prolongation was interpreted as conflict between the prior knowledge of color about traffic lights and the meaning of presented color [11]. These previous observations suggest that it is not the color itself, but the meaning of color that influences RTs.

In recent years, a driving simulator is often used as a tool for driving training. In the field of rehabilitation, the driving simulator has been reported to be effective in assessing and training the driving skills for dementia and stroke patients [15, 16, 17]. However, visual information presented on the simulator monitor is different from real traffic in terms of depth and texture, which possibly causes changes in the perception of environmental signals. In addition, simulation sickness during the driving simulation was found to be negatively correlated with the sense of presence, which is defined as the feeling of being an environment even if not physically present in that environment [18]. Thus, the environment in simulation could influence performance, and it is possible that a difference in visual modality (LED used in real traffic signal and monitor) influences RTs. Indeed, previous studies have reported that the processing pathway of visual information depends on the visual modality [19]. Therefore, it can be hypothesized that conflict between the prior knowledge of color and the meaning of presented color, causing a RT prolongation, can depend on the visual modality. By clarifying this, we may be able to provide valuable information that could contribute to the establishment of effective rehabilitation using different visual modalities.

The purpose of this study was to investigate whether a difference in visual modality influences the effect of prior knowledge of color on RTs. To this end, we evaluated RTs in a simple reaction task, a Blue Go/Red No-go task, and a Red Go/Blue No-go task, and compared them between two different signal presentation devices: an LED lighting device and a liquid crystal display (LCD) monitor.

## 2 Materials and methods

### 2.1 Participants

Fifteen healthy participants (7 females, mean age ±SD = 22.2 ± 2.5 years) took part in this study. All participants were right-handed as evaluated by the Edinburg Handedness Inventory [20], and had normal or corrected-to-normal vision. None of the participants had any special hobbies or backgrounds that would affect experimental results such as computer games. Written informed consent was obtained from all participants before beginning the experiment, which was performed according to principles of the Declaration of Helsinki. The study was approved by the Ethics Committee for Clinical Research of Hiroshima University (No. C-242).

### 2.2 Design and procedure

**Figure 1:**
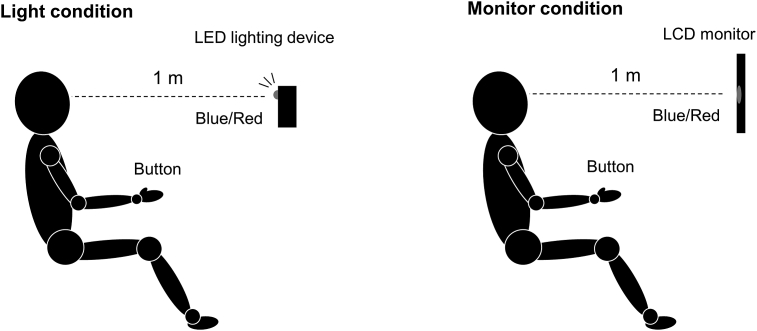
Schematic illustration of the experiment. The subject sat on a chair and performed a simple reaction task and a Go/No-go task with the right hand in the Light and Monitor conditions.

During the experiment, participants were seated on a comfortable reclining armchair with a mounted headrest. The signal presentation device was placed 1 m in front of them at eye level (Figure 1). Four reaction tasks were performed in a random order: a Blue simple reaction task, a Red simple reaction task, a Blue Go/Red No-go task, and a Red Go/Blue No-go task. Each task was performed using two different signal presentation devices: an LED lighting device (Light condition) and an LCD monitor (Monitor condition). In the Light condition, a custom-made LED lighting device (4 Assist, Tokyo, Japan) was used to present blue and red lights [21]. Red and blue LED bulbs were placed close to each other, and these lights were visible from one spot through a hole (5 mm) created in the device. In the Monitor condition, a red and blue circle with a diameter of 110 mm was displayed at the center of LCD monitor (32GK850F–B, LG electronics Japan, Tokyo, Japan), using a customized LabVIEW program (National Instruments, Texas, USA). A refresh rate, a gray-to-gray latency, and a pixel pitch of the LCD monitor were 144 Hz, 5.0 ms, and 0.2724 × 0.2724 mm, respectively. Since reaction time is different depending on the visual stimulus location (central or peripheral) [22, 23], the location of signal presentation was adjusted to the center of visual field in both conditions. The signal colors were set to blue and red according to the ISO standard. Luminance of red and blue signals in both conditions were measured using a luminance meter (HD2302.01, Delta OHM, Padova, Italy).

#### 2.2.1 Simple reaction task

Signals (duration of 100 ms) were presented for 100 times at a random interval of 1,500–1,800 ms. In both Light and Monitor conditions, there were two tasks, one for blue signal and one for red signal, which were conducted in a random order. The participants were instructed to react as fast as possible to the signal by pressing a button held in the right hand.

#### 2.2.2 Go/No-go task

In a Blue Go/Red No-go task, blue and red signals served as target (Go) and non-target (No-go) signals, respectively, while in a Red Go/Blue No-go task, red and blue signals served as target (Go) and non-target (No-go) signals, respectively. These two tasks were performed in a random order in both Light and Monitor conditions. Blue and red signals were randomly presented for a duration of 100 ms at a random interval of 1,500–1,800 ms. Signals were presented for 100 times in each task, and the Go probability was set as 30 % in all the tasks. The participants were instructed to react as fast as possible to the target (Go) signal by pressing a button held in the right hand and to withhold the response when a non-target (No-go) signal appeared.

### 2.3 RT recording and analysis

RT was defined as the interval between the signal onset and the onset of button press. Signals from the button presses, LED lighting device, and LCD monitor were all recorded using an analog-to-digital converter (PowerLab, AD Instruments, New South Wales, Australia), and stored in a personal computer for off-line analysis (LabChart 7, AD Instruments, New South Wales, Australia). RTs were calculated using MATLAB (MathWorks, Massachusetts, USA). Trials in which the participants did not respond to Go signals (Go omission errors) or responded to No-go signals (No-go commission errors) were excluded from the analysis of RTs. An error ratio was calculated as a ratio of the number of Go omission errors or No-go commission errors to the number of total signals in each Go/No-go task.

### 2.4 Statistical analysis

SPSS Statistics software version 21 (SPSS; IBM Corp., Armonk, NY, USA) was used for statistical analysis. We examined the distribution of data for normality using the Shapiro-Wilk test and confirmed that they were normally distributed (*p* > 0.05). A two-way repeated-measures analysis of variance (ANOVA) was used to determine the effect of Color (Blue and Red) and Device (Light and Monitor) on the mean RT in simple reaction and Go/No-go tasks. *Post-hoc* test was conducted with Bonferroni adjustment. Significant level was set at *p* < 0.05.

## 3 Results

The luminance of presented signals were 211.2 cd/m^2^ for blue and 63.7 cd/m^2^ for red in the Light condition, and 43.8 cd/m^2^ for blue and 31.3 cd/m^2^ for red in the Monitor condition.

**Figure 2:**
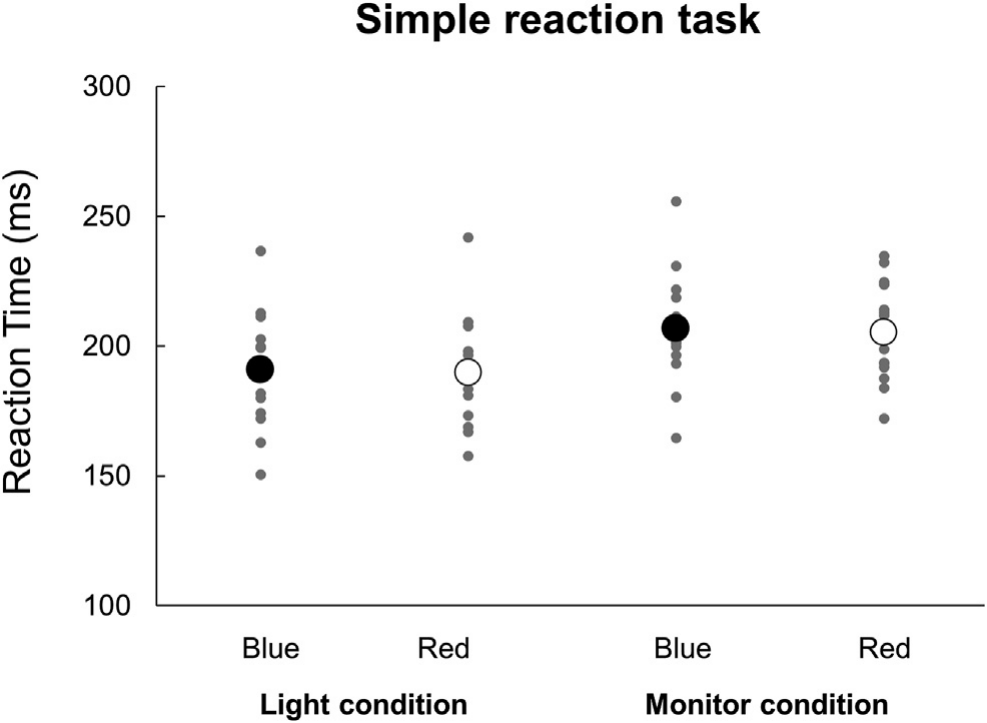
Reaction times in the simple reaction task. Individual data from all participants are presented for each condition. The black and white circles indicate the average. There was no significant difference between Blue and Red in both conditions.

**Figure 3:**
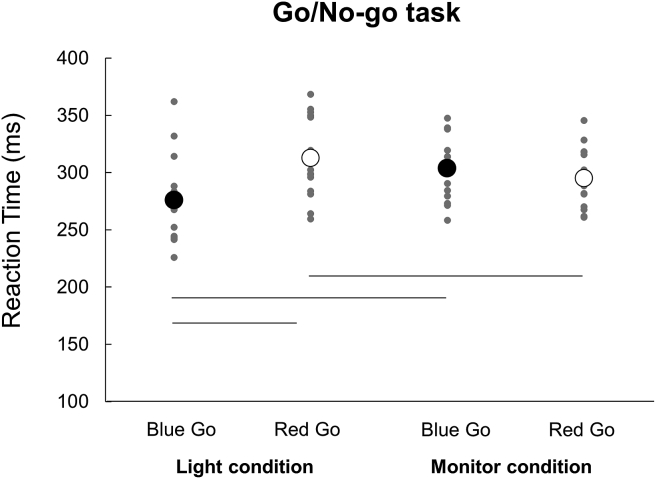
Reaction times in the Go/No-go task. Individual data from all participants are presented for each condition. The black and white circles indicate the average. The average reaction time was significantly longer in the Red Go/Blue No-go (Red Go) than Blue Go/Red No-go (Blue Go) task in the Light condition, but not in the Monitor condition.

The mean RTs for simple reaction and Go/No-go tasks are presented in Table 1. For RTs of a simple reaction task (Figure 2), a two-way repeated-measures ANOVA indicated a significant main effect of Device (*F* (1,56) = 16.534, *p* = 0.001, *η*^2^ = 0.541). There was no significant main effect of Color (*F* (1,56) = 0.481, *p* = 0.499, *η*^2^ = 0.033) or interaction between Color×Device (*F* (1,56) = 0.001, *p* = 0.98, *η*^2^ < 0.001). For RTs of a Go/No-go task (Figure 3), a two-way repeated-measures ANOVA indicated a significant main effect of Color (*F* (1,56) = 9.981, *p* = 0.007, *η*^2^ = 0.416) but not of Device (*F* (1,56) = 1.621, *p* = 0.224, *η*^2^ = 0.104). There was a significant interaction between Color×Device (*F* (1,56) = 34.832, *p* < 0.001, *η*^2^ = 0.713). *Post-hoc* analyses revealed that in the Light condition, the RT was significantly longer when responding to a red signal (Red Go/Blue No-go task) as compared to when responding to a blue signal (Blue Go/Red No-go task) (*p* < 0.05). On the other hand, in the Monitor condition, there was no significant difference in RT by signal color. Furthermore, the RT in response to a blue signal (Blue Go/Red No-go task) was significantly shorter in the Light than Monitor condition (*p* < 0.05). In addition, the RT in response to a red signal (Red Go/Blue No-go task) was significantly longer in the Light than Monitor condition (*p* < 0.05).

**Table 1:** Reaction times in simple reaction and Go/No-go tasks (ms).

	Simple reaction task (mean ± SD)		Go/No-go task (mean ± SD)	
	Blue	Red	Blue Go	Red Go
Light	191.1 ± 21.7	189.8 ± 20.5	276.1 ± 36.6	312.8 ± 34.8
Monitor	206.8 ± 21.1	205.4 ± 18.6	304.0 ± 26.9	295.2 ± 25.8

The mean error ratios are presented in Table 2. No statistical test was performed for the error ratios because most participants made no or few errors [21].

**Table 2:** Error ratios in Go/No-go task (%).

	Go/No-go task (mean ± SD)			
	Blue Go		Red Go	
	Go omission error ratio	No-go commission error ratio	Go omission error ratio	No-go commission error ratio
Light	0.0 ± 0.0	0.1 ± 0.4	0.0 ± 0.0	0.1 ± 0.4
Monitor	0.0 ± 0.0	0.1 ± 0.4	0.4 ± 1.7	0.1 ± 0.4

## 4 Discussion

The present study investigated whether a difference in visual modality influences the effect of prior knowledge of color on RTs. For that purpose, we evaluated RTs in a simple reaction task, a Blue Go/Red No-go task, and a Red Go/Blue No-go task, and compared them between two different signal presentation devices (Light condition and Monitor condition). As a result, in the simple reaction task, RTs to red and blue signal were similar in both conditions. On the other hand, in the Go/No-go task, RTs were significantly longer in the Red Go/Blue No-go than Blue Go/Red No-go task in the Light condition, but not in the Monitor condition.

In both Light and Monitor conditions, there was no difference in simple RTs to red and blue signals, as in a previous study [9]. This finding suggests that the perceptual processing of red and blue colors is similar when no cognitive load is present, regardless of the type of signal presentation modality. Thus, the difference in RT revealed for the Go/No-go tasks using LED is unlikely to be due to a mere difference in color, as described below.

In the Light condition, RTs were significantly longer in the Red Go/Blue No-go than Blue Go/Red No-go task. The cognitive process involved in the execution of Go/No-go tasks is motor inhibitory control to prevent unwanted responses. The decision is made in the frontal lobe, and the brain activity in this area has been shown to become larger with the increase in the cognitive load. For example, a functional magnetic resonance imaging study has reported a strong activation in the frontal association area and anterior cingulate cortex during a Go/No-go task [24]. A similar activation was also observed in the Stroop task [25] that requires the participants to judge whether the color and the meaning of a stimulus are congruent or incongruent [26, 27]. It was interpreted that this activation reflects the conflict detection and resolution processes. In our previous study, we revealed that the brain activity reflecting these processes was larger in Red Go/Blue No-go than Blue Go/Red No-go task [11]. Therefore, it is likely that a conflict between the prior knowledge of color and the meaning of presented color caused a prolongation of RT in the Red Go/Blue No-go task in the Light condition.

On the other hand, in the Monitor condition, there was no difference in RT between the Blue Go/Red No-go and Red Go/Blue No-go tasks. This result likely indicates that a conflict between the prior knowledge of color and the meaning of presented color was not sufficient to change RTs, unlike the Light condition. Although a precise mechanism of this phenomenon cannot be explained, we hypothesize that blue and red signals presented from the LCD monitor were insufficient to evoke a perception of traffic lights as compared to the LED. When responding to visual stimuli, visual information (retinal activity) is processed through two different pathways in the visual system [28]. Information about direction of motion and depth is processed by magnocellular layer in the lateral geniculate nucleus and travels through the dorsal pathway to the parietal lobe after reaching the visual cortex. The processing pathway has primarily been studied in the macaque monkey brain, where single neuron recordings can be performed [29]. On the other hand, information about color and shape is processed by parvocellular layer in the lateral geniculate nucleus and travels through the ventral pathway to the temporal lobe after reaching the visual cortex [24, 25]. Hence, an object is recognized in the temporal lobe, especially in the inferior temporal cortex [30] that selectively perceives three-dimensional (3D) rather than two-dimensional (2D) information [31]. In addition, it has been reported that information from the dorsal pathway travels from the posterior parietal cortex to the inferior temporal cortex [32], suggesting that information such as depth could affect the object recognition. Indeed, a previous study has demonstrated that visual information processing performance was poorer with virtual reality images (2D) than real-world images (3D) [33]. In the Monitor condition of the present study, the signal was 2D, and thus its depth and texture were different from the real traffic lights. Accordingly, it is possible that this difference (the lack of 3D information) has caused a decrease in the level of perception of the presented signals as traffic lights, resulting in less conflict between the prior knowledge of color and the meaning of presented color. Based on our findings, it may be interesting to conduct research using tasks that resemble actual driving environments using virtual reality and driving simulators. Previous studies have suggested that game-like tasks using virtual reality can be motivating for all ages, and that games can have more pronounced training effects and less variability in response [34]. Furthermore, not only traffic signals and traffic signs but also other objects that exist in reality, such as pedestrians and vehicles, can be produced in the game-like tasks. However, RTs to signals can be influenced by the type of visual modality (Monitor vs. LED); thus, data from the game-like tasks may need to be interpreted with caution, and hence further research would be required in this aspect.

Finally, we would like to consider how our findings can be translated to clinical application. From our findings, it can be predicted that reaction to a red signal is slower during driving on the real road (Light condition) than during driving on a simulator (Monitor condition). This may cause a dissociation between the car driving ability evaluated using the driving simulator and the true driving ability on the road. Therefore, we need to take this dissociation into account when providing rehabilitation for driving. For example, it would be important to practice to press a brake pedal in response to a red signal at earlier timing during a driving simulator training in consideration with slow reactions to 2D traffic lights, and/or to press a brake pedal quickly in response to a red LED light. However, in the present study the participants responded to signals by pressing a button held in the hand. Therefore, it would be necessary to investigate whether our findings can be confirmed in a task that requires participants to press a foot pedal in response to a signal in the future.

There are limitations that should be acknowledged in this study. First, the sample size was small. Thus, the null result (Monitor condition) should be interpreted with caution. Second, the luminance of presented stimulus was not completely equal between the colors and between the conditions. It has been reported that RTs to visual stimuli are affected by their luminance in a simple reaction task [35]. Although there was no difference in simple RTs to red and blue signals in both Light and Monitor conditions in the present study, we cannot completely rule out a possibility that differences in the luminance and other cofounds related to the stimuli have influenced our results. Third, we did not measure neurophysiological data, necessitating a functional brain imaging study to confirm whether activity in the frontal association area, anterior cingulate cortex, and temporal lobe differs depending on the visual modality.

## 5 Conclusion

We found that a prolongation of RT due to conflict between the prior knowledge of color and the meaning of presented color in a Go/No-go task occurred when signals were presented by an LED lighting device, but not by an LCD monitor. The results of this study suggest that a difference in the presentation modality of visual information can influence the level of object perception and consequently the effect of prior knowledge on behavioral responses.

## Declarations

### Author contribution statement

Takayuki Horinouchi: Performed the experiments; Analyzed and interpreted the data; Contributed reagents, materials, analysis tools or data; Wrote the paper.

Tatsunori Watanabe: Conceived and designed the experiments; Wrote the paper.

Takuya Matsumoto: Performed the experiments; Analyzed and interpreted the data; Contributed reagents, materials, analysis tools or data.

Keisuke Yunoki, Takayuki Kuwabara, Kanami Ito, Haruki Ishida: Analyzed and interpreted the data; Contributed reagents, materials, analysis tools or data.

Hikari Kirimoto: Conceived and designed the experiments; Wrote the paper.

### Funding statement

This work was partially supported by the 10.13039/501100001691Japan Society for the Promotion of Science (19H01091, 19H03977, and 20K19708).

### Data availability statement

Data included in article/supplementary material/referenced in article.

### Declaration of interests statement

The authors declare no conflict of interest.

### Additional information

No additional information is available for this paper.
